# P51 - ADRB2 gene polymorphisms of the asthma pediatric patients in Russia’s Perm region

**DOI:** 10.1186/2045-7022-4-S1-P106

**Published:** 2014-02-28

**Authors:** Evgenii Furman, Mariya Ponomareva, Yurii Pechenkin, Irina Koryukina

**Affiliations:** 1 Perm State Medical Academy, Perm, Russia

## 

B2 adrenergic receptor (ADRB2) gene variation could explain differences in bronchodilator response among patients with asthma or identify a subgroup of patients with reduced response.

The aim of our research was identified polymorphisms in the ADRB2 gene in Russian asthma pediatric patients (in Perm region).

44 children from 3 to 18 years of age with bronchial asthma were examined. Mild asthma was in 95,5% , moderate asthma – in 4,5%. Gly16Arg and Gln27Glu mutations of ADRB2 gene were identified by PCR technique.

## Results

Gly16Arg mutations of ADRB2 gene was identified in 45,5% and Gln27Glu mutations – in 27,2% (table 1).

**Table 1:** The distribution of genotypes of ADRB2 gene in children with asthma

Variant	Genotipe					
**rs1042713**	**AA**		**AG**		**GG**	

	%	n	%	n	%	n

	18,1	8	36,4	16	45,5	20

**rs 1042714**	**CC**		**CG**		**GG**	

	36,4	16	36,4	16	27,2	12

There were not correlations between severity of bronchial asthma and ADRB2 gene polymorphisms (Gly16Arg and Gln27Glu mutations). The total IgE level was increased in group with Gly16Arg mutation 565,6±242,6 ME/ml, comparatively group without mutation (255±60,31 ME/ml, p=0,563). We found that asthma exacerbations in children with gene mutations occurs after allergen’s exposure in 64% cases. In group without the mutation asthma exacerbations occurs in acute viral respiratory infections in 68% cases.

Thus ADRB2 gene polymorphisms may be associated with clinical features of asthma in children.

